# HTPheno: An image analysis pipeline for high-throughput plant phenotyping

**DOI:** 10.1186/1471-2105-12-148

**Published:** 2011-05-12

**Authors:** Anja Hartmann, Tobias Czauderna, Roberto Hoffmann, Nils Stein, Falk Schreiber

**Affiliations:** 1 Leibniz Institute of Plant Genetics and Crop Plant Research (IPK), Corrensstrasse 3, 06466 Gatersleben, Germany; 2 Martin Luther University Halle-Wittenberg, Institute of Computer Science, Von-Seckendor-Platz 1, 06120 Halle, Germany

## Abstract

**Background:**

In the last few years high-throughput analysis methods have become state-of-the-art in the life sciences. One of the latest developments is automated greenhouse systems for high-throughput plant phenotyping. Such systems allow the non-destructive screening of plants over a period of time by means of image acquisition techniques. During such screening different images of each plant are recorded and must be analysed by applying sophisticated image analysis algorithms.

**Results:**

This paper presents an image analysis pipeline (HTPheno) for high-throughput plant phenotyping. HTPheno is implemented as a plugin for ImageJ, an open source image processing software. It provides the possibility to analyse colour images of plants which are taken in two different views (top view and side view) during a screening. Within the analysis different phenotypical parameters for each plant such as height, width and projected shoot area of the plants are calculated for the duration of the screening. HTPheno is applied to analyse two barley cultivars.

**Conclusions:**

HTPheno, an open source image analysis pipeline, supplies a flexible and adaptable ImageJ plugin which can be used for automated image analysis in high-throughput plant phenotyping and therefore to derive new biological insights, such as determination of fitness.

## Background

High-throughput analysis methods are commonly used in molecular biology. Recently, high-throughput phenotyping has been introduced to capture phenotypical data in larger quantities. Automated greenhouses, in which plants are grown and analysed automatically and images are taken in regular intervals, are the basis for high-throughput phenotyping for plants. Image analysis software augments an observer's ability to evaluate plant phenotypes.

Plants are the main source of human nutrition, increasingly meaningful for renewable resources and can provide ingredients that provide health benefits. Plant research deals with questions such as which genetic information is responsible for the plants' characteristics in order to gain high yield, or stability despite the global warming and other stress situations.

To improve plant-breeding, numerous experiments for large plant populations grown under strictly controlled environmental conditions (such as water availability, continuous lighting- and temperature conditions) are conducted. To determine the performance and the tolerance to different biotic and abiotic environmental conditions (e. g. quantification of the sensitivity to drying stresses) phenotypes should be analysed non-invasively by imaging throughout their growth cycle.

**Figure 1:**
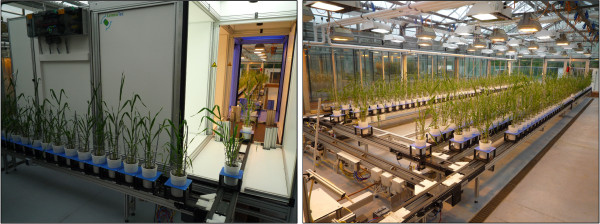
**View of the automatic greenhouse system**. Image acquisition device for images in visible, near-infrared and ultra-violet spectra (left), greenhouse device consisting of a conveyor belt system carrying 312 barley plants (right).

For this purpose various fully automatic high-throughput plant growth and phenotyping platforms have been developed. A technology called PHENOPSIS [1] developed by Optimalog is used by the French National Institute for Agricultural Research (INRA) for *Arabidopsis thaliana*. The Research Center Jülich analyses phenotypes of different plant species with the in-house GROWSCREEN system [2]. Both techniques use a camera which is moved over the plants. The company CropDesign developed the TraitMill™ platform [3], a high-throughput technology that enables large-scale transgenesis and plant evaluation of *Oryza sativa*. This and the high-throughput phenotyping platform developed by LemnaTec [4] are fully automatic greenhouse systems that screen plants non-destructively over a period of time. LemnaTec systems are, for example, used at the Australian Centre for Plant Functional Genomics (ACPFG) and the Leibniz Institute of Plant Genetics and Crop Plant Research (IPK) in Gatersleben. As an example, the system at IPK Gatersleben allows the growth of barley plants in 312 pots under controlled environmental conditions. Each plant is located in a carrier which is situated upon a conveyor belt. The conveyor belt system automatically retrieves each plant as needed and passes it through the image capture units (see Figure 1). In this system, plants are captured autonomously in near infrared-, ultra violet- and visible spectra in three boxes. Each box contains a top view camera and a side view camera and furthermore a turnable lifter which enables the plants to be lifted and turned. After imaging, plants pass the watering and weighing unit, which automatically measures weights and waters the plants. Since each carrier is tagged with an RFID chip each plant can be identified and traced during its growth cycle. An extensive amount of data is generated by the platform within a period of plant development. This data is stored in a database system.

High-throughput image analysis for automated phenotyping is used to extract several phenotypic parameters related to growth, yield and stress tolerance of the plants. In this manner the tedious and time-consuming manual analysis of many phenotypes is reduced. However, no freely available, open-source software for high-throughput image analysis is available, and commercial systems are limited. For example, the LemnaTec system is delivered with the commercial software LemnaGrid. This proprietary software with its built-in algorithms cannot easily be modified if desired, except for a limited set of parameters, and results of the standard pipeline for new plant species are often unsatisfactory.

**Figure 2:**
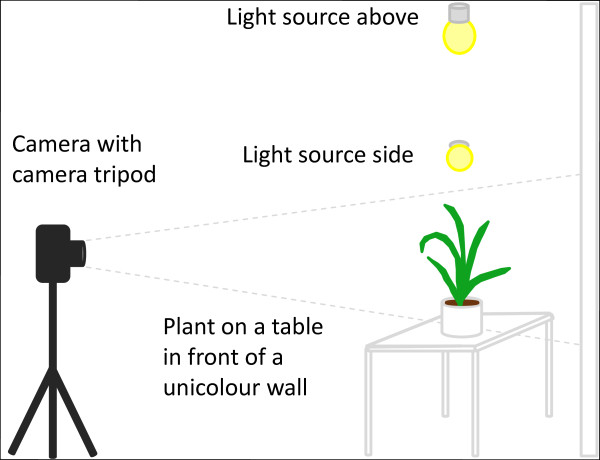
**Possible manual system for image acquisition**. The light sources from above and both sides avoid shadows on leaves.

There exists a clear need for robust, flexible and step by step traceable image analysis tools for plant phenotyping. In this study, we describe the ImageJ [5] plugin HTPheno, which is a freely available open source pipeline to handle image data from plants. It has been developed in a modular way to allow the analysis of images from different phenotyping sources. HTPheno has been tested with the LemnaTec system, as other phenotyping platforms were not available to the authors. However HTPheno is a generalized image analysis pipeline which can be adapted by the user to other phenotyping systems and therefore be used by a wide community of plant scientists. It is also possible to record the images independent from automated phenotyping systems which acquire all images of the individual plants. If a laboratory would like to use the HTPheno image processing pipeline the user could acquire images in a manual fashion as shown in Figure 2.

Such a manual image acquisition consists of a commercially available standard camera with a camera tripod which is positioned in some distance to the plant to reduce perspective distortion. Depending on the lens of the camera and its resolution the perspective distortion could also be reduced by a preprocessing step in the image analysis pipeline. The plant should be arranged on a table which is in front of a unicolour wall. In our experiments light blue showed a good colour choice as it is easily separable from colours in the plants. For an adequate illumination it is recommended to install a light sources above and on both sides of the plant. This setup enables the user to record images from different plants manually. Images from such a manual and low cost system can also be the source for computation of phenotypic parameters by HTPheno. To enable a versatile applicability of HTPheno to different high-throughput phenotyping setups, we decided to follow a modular approach: several configuration files allow the adjustment of the plugin to the users' needs. With the HTPheno plugin it is possible to retrieve single images or a series of images from the local file system and to automatically analyse the coloured images via colour segmentation. The phenotypic data is determined on the basis of the segmented plant: for the side view it is plant height, width and projected shoot area; for the top view it is x-extent, y-extent, projected shoot area and the diameter of the plant.

Images are finally illustrated in an image stack and the phenotypic data are composed in a result table which can be exported into various spreadsheet applications to derive meaningful diagrams.

## Implementation

### Image analysis pipeline HTPheno

There exist several tools supporting image editing, image processing and image analysis for many biological applications [6]. One popular tool is ImageJ [5], a flexible, open source image processing software based on Java. It comes with a graphical user interface and, with regard to scientific analysis, a collection of useful plugins and tools. Besides platform independence, the main reason for the wide distribution of the ImageJ software is the extensibility via Java plugins.

**Figure 3:**
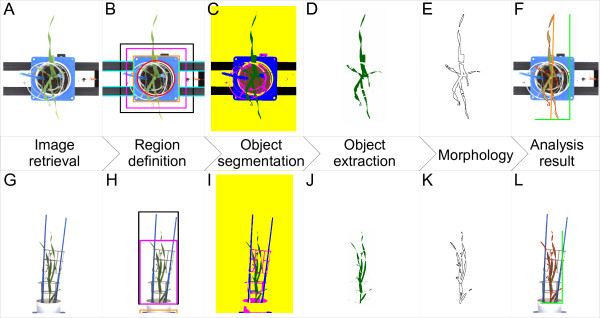
**High-throughput image analysis pipeline HTPheno for top view (A-F) and side view (G-L) images**. From left to right: 1) retrieve images (A, G). 2) define regions for top view (B): region of soil (red circle), carrier (orange rectangle), cages (magenta rectangle), sticks (black rectangle) and conveyor belt (light blue rectangles) respectively side view (H): carrier (orange rectangle), cages (magenta rectangle) and sticks (black rectangle). 3) segment defined objects by colour segmentation (C, I): soil (brown), carrier and sticks (blue), cages (magenta), conveyor belt (black), background (yellow) and the plant (green). 4) Object extraction (D, J). 5) morphological opening (E, K). 6) analysis result (F, L) showing outline of plant (red), in top view x-extent, y-extent (light green), and diameter (orange) of plant, in side view width and height (light green) of plant.

HTPheno is such a plugin and provides an adaptable image analysis pipeline for high-throughput phenotyping. With two built-in functions, (1) the calibration (HTPcalib) to specify different parameters for segmentation and (2) the automatic image processing, it can be used for analysing colour images in side view and top view. The HTPheno plugin realises automatic image processing for a number of images involving steps such as: region definition, object segmentation, display of the object extraction, morphological operation and compilation of the analysis results (see Figure 3). Finally, analysis results for all plants are comprised in a table and processing steps for each plant are visualised in an image stack.

### Calibration of parameters for segmentation

Segmentation, which specifies if a pixel belongs to a defined object or not, is the essential and critical step in image processing. A colour image segmentation approach consists of the monochrome segmentation approach operating in different colour spaces. Some commonly used monochrome segmentation approaches are: histogram thresholding, feature space clustering, region based approaches, fuzzy approaches, neural networks or physics based approaches. They can operate in, for example, the following colour spaces: RGB, YIQ, YUV, I_1_I_2_I_3_, HSV, Nrgb, CIE L*u*v* or CIE L*a*b* [7]. Two fundamental questions arise in this context:

• Which colour space is suitable for the recorded images?

• Which segmentation method achieves a precise segmentation of the plant?

The colour image segmentation approach used here is multidimensional histogram threshholding: the histogram thresholding in combination of the two colour spaces RGB and HSV, which are appropriate colour spaces for images recorded by the high-throughput phenotyping platform.

#### Colour space

Based on the high correlation among the three primary colours red (R), green (G) and blue (B) colour image segmentation poses a challenge. If the brightness of the image changes due to changing or instable light conditions, all three colour components change accordingly. Hence we decided to use additionally the HSV colour space which is more intuitive to human perception. It separates colour information from brightness information. The human vision system can distinguish different basic colours (H, hue) easily, whereas the change of brightness (V, value) or purity of colour (S, saturation) does not imply the recognition of different colours [7].

By using nonlinear transformation the HSV colour space can be derived from RGB colour space. This means a linear change in H, S and V does not result in a linear change of RGB parameters. Therefore a slight change of input R, G, and B values can cause a large jump in the transformed H, S, and V values. Due to nonlinear transformation Hue has a non removable singularity and is numerically unstable at low saturation. If the intensity of the colour is close to white or black, Hue and Saturation play little role in distinguishing colours [7]. The HSV colour space alone is not sufficient for the segmentation of images recorded by the LemnaTec facility. Using a combination of RGB and HSV colour space results in a strong correlation of the calculated parameters obtained by HTPheno with the manually measured values (see section Validation).

#### Segmentation method

The irregular morphology of plants (here barley, *Hordeum vulgare*) restricts the development of a simple model which is at least necessary for the physics based segmentation approach. Images from the high-throughput phenotyping platform often have non-uniform illumination with the consequence of colour similarity between objects (such as carrier, conveyor belt, cages, sticks and shadows of them) and inhomogeneity within one object. Hence tests with an automatic segmentation method, the ImageJ plugin multi otsu threshold [8], an implementation of the otsu threshold algorithm to find up to 5 optimal threshold level (multilevel) of an image [9], does not deliver the required thresholds.

The best choice under these preconditions is to use a pixel based segmentation approach called multidimensional histogram thresholding (MHT). It utilises gray values of pixels without considering the neighbourhood. An image consists of areas in different gray level ranges. These areas can be separated in the histogram of the image by means of thresholds. Applied to colour images this approach operates in each colour channel histogram. An object is thus defined by a minimal and a maximal threshold for every channel of the RGB colour space and HSV colour space. To easily determine the thresholds for the object segmentation the function HTPcalib was developed. Images recorded by the high-throughput phenotyping platform contain beside the plant other objects. The user defines these objects by assigning them the correct colours in the image.

Some objects have a colour similar to the plant. Using this segmentation approach they would be segmented as plant as well. Therefore segmentation takes place in user-defined regions for top view images and side view images. A known object can only be situated within its defined region. Hence a set of regions can be defined. Here five regions are defined for top view images (region of soil, carrier, cages, sticks, and conveyor belt) and three regions are defined for side view images (region of carrier, cages, and sticks) since the cameras are installed in a fixed position (see Figure 3B, H). Once defined, regions grow and shrink automatically dependent on a user-defined scaling factor which depends on camera settings. Also a factor to translate pixel size into millimeter is set in HTPcalib.

After completing the calibration the automatic image processing for top view images and side view images can be applied.

### Image processing

HTPheno retrieves single or series of images from the local file system and analyses automatically these images. Each processing step is visualised by an image (see Figure 3) first the original image is loaded (see Figure 3A, G), then the defined regions of the image are added (see Figure 3B, H) and after applying the object segmentation (MHT) a colour-coded image is shown (see Figure 3C, I).

Before performing the next step in the analysis pipeline the object of interest (the plant) is extracted (see Figure 3D, J). The plant has no defined region since plant parts can be located anywhere in the image. Some incorrectly segmented pixels and regions of the plant may occur because of colour similarity. To improve the segmentation of the plant the morphological operation opening is applied. Opening solves the problem by performing erosion followed by dilation. Opening removes small objects from the foreground (usually taken as dark pixels) of an image, placing them in the background and then smoothes objects.

The resulting refined segmented plant has a lower noise level (see Figure 3E, K).

Finally calculations based on the morphology result are performed. The visual analysis result is transferred to the original image and consists of plant outline; plant x-extent, plant y-extent and plant diameter (top view); plant width and plant height (side view). To get an impression of the plant size a scale bar (100 mm) is added to the image in the bottom right corner. All analysis steps can be checked by direct comparison of processed and non-processed images in an image stack per plant. A result table comprises all obtained phenotypic data: x-extent, y-extent and diameter in top view, width and height in side view as well as projected shoot area in both views for all plants. This result table can be exported into various spreadsheet applications for further processing.

## Results

Analysing images from high-throughput screening experiments is time consuming and computationally demanding. High-throughput screening facilities produce thousands of images of plants per day and researchers are currently limited by the lack of open source software for automated high-throughput image analysis. The HTPheno plugin for ImageJ [5] is such an automated high-throughput software to analyse images and to detect characteristics of different plant phenotypes.

The tool analyses phenotypic parameters in a much shorter period of time than manual measurements. For example, measuring 8 plants at 6 different time points in side view and top view manually means to measure parameters from 96 images. Obtaining parameters such as height and width in side view images and x-extent, y-extent and diameter in top view images takes 4 hours of manual work. Using HTPheno the same task can be accomplished in less than one minute on a state of the art desktop PC. During this process the software additionally calculates the projected shoot area. A manual measurement of this parameter implies great effort and needs more time.

### Validation

**Figure 4:**
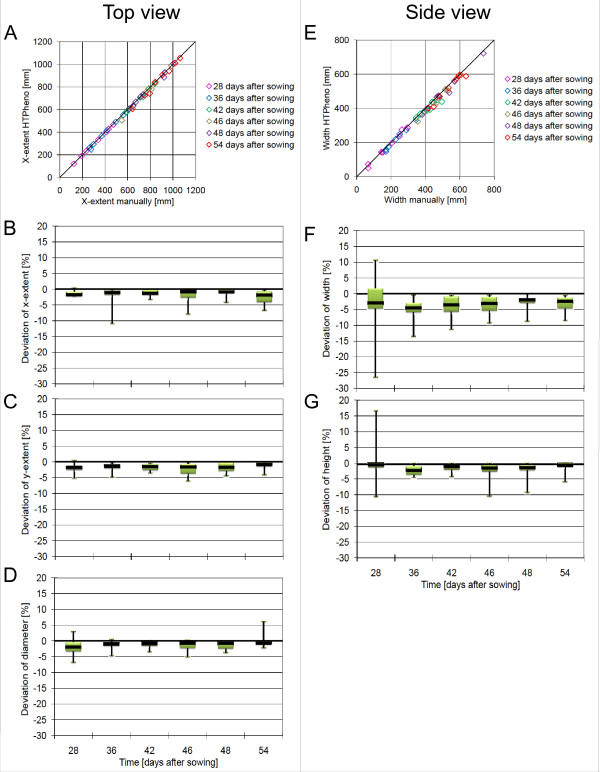
**Validation of HTPheno**. Two exemplary correlations (A, E) and the deviation of automatically obtained values from manually measured values are plotted. The values have been manually derived from the images of 8 plants: top view (A, B, C, D), side view (E, F, G) at 6 different days after sowing. The colours in the correlation diagrams (A, E) represents 6 different time points after sowing. Every bar in the deviation diagrams shows the mean deviation of the automatically obtained values from the manually obtained values for these 8 plants as black line and the interquartile range between the lower quartile and the upper quartile in green which indicate the distribution of 50 percent of the samples. Additionally the range (minimal and maximal parameter values) is given as black horizontal line.

To validate the results produced by the HTPheno plugin images of 8 plants were chosen from an experiment period which started at day 28 after sowing and ended at day 54 after sowing. Different parameters were measured manually for comparison with the obtained parameters from the HTPheno plugin (see Figure 4). The x-extent values in top view images and the width values in side view images obtained by HTPheno correlate strongly with the manually measured x-extent values and width values (see Figure 4A, E).

The mean values of all obtained parameters by HTPheno deviate slightly from the manually measured parameters. The x-extent, y-extent and diameter in top view (see Figure 4B, C, D) and the width and hight in side view (see Figure 4F, G) are smaller than the manually obtained values. Due to colour similarity between objects and the plant itself and because of shadows and reflexions HTPheno currently cannot detect yellow and brown parts of the plant such as leaf tips. If these colours are defined as plant it may occur that parts of the soil and the cages are detected as plant parts too. Therefore yellow and brown tones are currently excluded from the measurements performed by HTPheno. This leads to an offset of the interquartile range below the base line and also to outliers. Investigating the analysis results the outliers were particularly caused by leaf tips and brown and yellow leaf parts which could not be segmented as plant.

Nevertheless there exists an outlier at day 28 after sowing in the deviation of width and height in the side view image above the base line. For example, the obtained width deviates more than 10 percent from the manually measured value. An investigation of the analysis results shows that reflexions at the pot and at a screw of the carrier are also segmented as plant. Since this plant is very small at this developmental stage the deviation seems to be large. However, the absolute difference is small, the value calculated by HTPheno is 73 mm and the manually measured value is 66 mm.

Except for these outliers all parameters obtained by HTPheno are in agreement with the manually measured parameters. Altogether HTPheno provides good results for the analysis of colour images taken from side view and from top view.

### Application example

**Figure 5:**
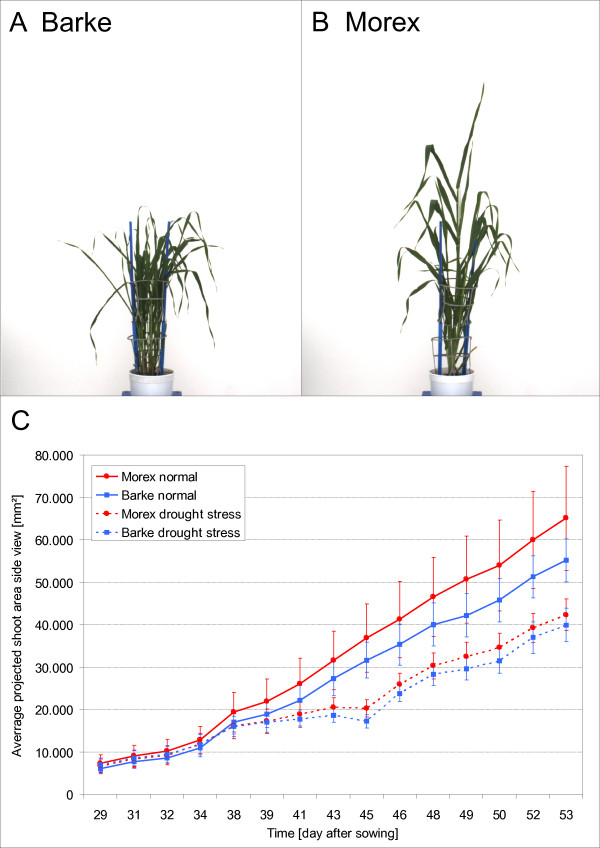
**Comparison of different barley (Hordeum vulgare) cultivars**. Habitus of cultivar *Barke *and of cultivar *Morex *in side view (A, B). Plot of the average projected shoot area in side view of 312 barley plants composed of the two cultivars with 78 *Barke *plants and 78 *Morex *plants under well watered conditions (solid line) and 78 *Barke *plants and 78 *Morex *plants under drought stressed conditions (dashed line). Both cultivars have lesser average projected shoot area under drought stress conditions but approximate to plants under well watered conditions in the last days of the experiment (C). The average projected shoot area of cultivar *Morex *is under both conditions larger. Vertical bars show ± S.E. of 78 replicates. Analysis was carried out with HTPheno.

The application example shown in Figure 5 represents the comparison of different barley (*Hordeum vulgare*) cultivars. Both cultivars *Barke *and *Morex *are precisely defined homozygous genotypes. *Barke *plants are identical among each other and *Morex *plants are identical among each other, but *Barke *and *Morex *do not correspond. They are 99.9 percent genetically identical, but vary widely in morphological characteristics. The habitus of cultivar *Barke *has semi-dwarfed growth and develops thin leaves. To compensate the semi-dwarfed growth *Barke *plants tiller (see Figure 5A). *Morex *plants by contrast grow higher and develop leaves with larger area (see Figure 5B).

To compare the fitness of these two cultivars with different treatments over a period of time the phenotyping platform took side view images of 78 plants per condition and cultivar. 78 of 156 *Barke *plants and 78 of 156 *Morex *plants were exposed to drought stress, the remaining plants were well watered during the experiment period. In this experiment images were taken from day 29 after sowing until day 53 and the drought stress period started at day 30 and ended at day 45. All images were analysed by HTPheno to obtain phenotypic parameters such as width, height and projected shoot area. The growth rate over a period of time measured at 15 time points is illustrated by average values of all plants per cultivar and condition and the standard deviation (see Figure 5C).

The average projected shoot area of cultivar *Morex *is under both conditions larger. Due to its morphology *Morex *is tall and has widespread leaves occupying a great area of the images. *Barke *tillers more and has many thin overlapping leaves occupying a smaller area of the images than *Morex*. The standard deviation of *Morex *under normal conditions is much higher than the one for *Barke*. Investigating the images it was observed that *Morex *plants develop differently whereas *Barke *plants develop more similarly. Both cultivars have a smaller average projected shoot area under drought stress conditions.

The image analysis shows that the phenotypic parameter projected shoot area can be used to describe morphological differences between the two barley cultivars *Barke *and *Morex *as well as differences in growth under different conditions.

## Conclusions

In this paper HTPheno, a novel open source image analysis pipeline for high-throughput plant phenotyping, has been presented. This ImageJ plugin provides the possibility to automatically analyse the colour images taken from side view and from top view. With the calibration function HTPcalib parameters of HTPheno can be adapted to analyse other plant species recorded by a high-throughput phenotyping platform. In the application example we have shown that HTPheno is a useful plugin for measuring phenotypic parameter, such as projected shoot area in top and side view of plants to distinguish different phenotypes.

We hope to inspire ideas within the phenotyping community for further development. Researchers are welcome to download the Java source code http://htpheno.ipk-gatersleben.de/ and add new analysis tools to the system.

## Availability & Requirements

Project Name: HTPheno

Project Home Page: http://htpheno.ipk-gatersleben.de/

Programming Language: Java

Other Requirements: Java 5 or higher; ImageJ

## Authors' contributions

The software was designed and written by AH and RH. FS created the software concept. NS designed the biological study. AH, TC and FS evaluated the results. AH drafted the manuscript. All authors finalised and approved the manuscript.

## References

[B1] Granier C Aguirrezabal L Chenu L Cookson SJ Dauzat M Hamard P Thioux JJ Rolland G Bouchier-Combaud S Lebaudy A Muller B Simonneau T Tardieu F PHENOPSIS, an automated platform for reproducible phenotyping of plant responses to soil water deficit in Arabidopsis thaliana permitted the identification of an accession with low sensitivity to soil water deficit New Phytologist 2006 169 623 635 10.1111/j.1469-8137.2005.01609.x 16411964

[B2] Walter A Scharr H Gilmer F Zierer R Nagel KA Ernst M Wiese A Virnich O Christ MM Uhlig B Juenger S Schurr U Dynamics of seedling growth acclimation towards altered light conditions can be quantified via GROWSCREEN: a setup and procedure designed for rapid optical phenotyping of different plant species New Phytologist 2007 174 447 455 10.1111/j.1469-8137.2007.02002.x 17388907

[B3] Reuzeau C Pen J Frankard V de Wolf J Peerbolte R Broekaert W van Camp W TraitMill™: a Discovery Engine for Identifying Yield-enhancement Genes in Cereals Molecular Plant Breeding 2005 5 753 759

[B4] LemnaTec - Imageprocessing in Biology http://www.lemnatec.com

[B5] ImageJ - Image Processing and Analysis in Java http://rsbweb.nih.gov/ij/

[B6] Walter T Shattuck DW Baldock R Bastin ME Carpenter AE Duce S Ellenberg J Fraser A Hamilton N Pieper S Ragan MA Schneider JE Tomancak P Hériché JK Visualization of image data from cells to organisms Nature methods 2010 7 S26 S41 10.1038/nmeth.1431 20195255 PMC3650473

[B7] Cheng HD Jiang XH Sun Y Wang JL Color image segmentation: Advances and prospects Pattern Recognition 2001 34 2259 2281 10.1016/S0031-3203(00)00149-7

[B8] Tosa Y Multi Otsu Threshold http://rsbweb.nih.gov/ij/plugins/multi-otsu-threshold.html

[B9] Liao P Chen T Chung P A fast algorithm for multilevel thresholding Journal of Information Science and Engineering 2001 17 713 727

